# COVID-19 in Female and Male Athletes: Symptoms, Clinical Findings, Outcome, and Prolonged Exercise Intolerance—A Prospective, Observational, Multicenter Cohort Study (CoSmo-S)

**DOI:** 10.1007/s40279-023-01976-0

**Published:** 2024-01-11

**Authors:** Manuel Widmann, Roman Gaidai, Isabel Schubert, Maximilian Grummt, Lieselotte Bensen, Arno Kerling, Anne Quermann, Jonas Zacher, Shirin Vollrath, Daniel Alexander Bizjak, Claudia Beckendorf, Florian Egger, Erik Hasler, Klaus-Peter Mellwig, Cornelia Fütterer, Fritz Wimbauer, Azin Vogel, Julia Schoenfeld, Jan C. Wüstenfeld, Tom Kastner, Friedrich Barsch, Birgit Friedmann-Bette, Wilhelm Bloch, Tim Meyer, Frank Mayer, Bernd Wolfarth, Kai Roecker, Claus Reinsberger, Bernhard Haller, Andreas M. Niess, CoSmo-S Consortium, Mike Peter Birnbaum, Christof Burgstahler, Michael Cassel, Peter Deibert, Katrin Esefeld, Gunnar Erz, Franziska Greiss, Martin Halle, Judith Hesse, Karsten Keller, Christine Kopp, Lynn Matits, Hans Georg Predel, Peter Rüdrich, Gerald Schneider, Philipp Stapmanns, Jürgen Michael Steinacker, Sarah Szekessy, Andreas Venhorst, Stephanie Zapf, Christian Zickwolf

**Affiliations:** 1 Department of Sports Medicine, Medical Clinic, Medical University Hospital Tuebingen, University Hospital of Tuebingen, Hoppe-Seyler Str. 6, 72076 Tuebingen, Germany; 2 Department of Sports and Health, Institute of Sports Medicine, Paderborn University, Paderborn, Germany; 3 Department of Sports Medicine, Charité-Universitätsmedizin Berlin and Humboldt-Universität zu Berlin, Berlin, Germany; 4 Clinic for Rehabilitation and sports medicine, Hannover Medical School, Hannover, Germany; 5 Medical Clinic VII, Department of Sports Medicine, University Hospital Heidelberg, Heidelberg, Germany; 6 Department of Preventative and Rehabilitative Sports and Performance Medicine, Institute of Cardiology and Sports Medicine, German Sports University Cologne, Cologne, Germany; 7 Division of Sports and Rehabilitation Medicine, Ulm University Medical Center, Ulm, Germany; 8 Center of Sports Medicine, Outpatient Clinic, University of Potsdam, Potsdam, Germany; 9 Institute of Sports and Preventive Medicine, Saarland University, Saarbrücken, Germany; 10 Institute for Applied Health Promotion and Exercise Medicine (IfAG), Furtwangen University, Furtwangen, Germany; 11 Clinic for General and Interventional Cardiology/Angiology, Herz-und Diabeteszentrum NRW, Ruhr-Universität Bochum, Bad Oeynhausen, Germany; 12 School of Medicine, Institute of AI and Informatics in Medicine, Technical University of Munich, Munich, Germany; 13 Department of Prevention and Sports Medicine, University Hospital ‘Rechts Der Isar’, Technical University of Munich, Munich, Germany; 14 Medical Faculty, Institute of Exercise and Occupational Medicine, University Freiburg, Freiburg, Germany; 15 Institute for Applied Training Science, Leipzig University, Leipzig, Germany; 16 Department of Molecular and Cellular Sport Medicine, Institute of Cardiovascular Research and Sport Medicine, German Sport University Cologne, Cologne, Germany

## Abstract

**Background:**

An infection with SARS-CoV-2 can lead to a variety of symptoms and complications, which can impair athletic activity.

**Objective:**

We aimed to assess the clinical symptom patterns, diagnostic findings, and the extent of impairment in sport practice in a large cohort of athletes infected with SARS-CoV-2, both initially after infection and at follow-up. Additionally, we investigated whether baseline factors that may contribute to reduced exercise tolerance at follow-up can be identified.

**Methods:**

In this prospective, observational, multicenter study, we recruited German COVID elite-athletes (cEAs, *n* = 444) and COVID non-elite athletes (cNEAs, *n* = 481) who tested positive for SARS-CoV-2 by PCR (polymerase chain reaction test). Athletes from the federal squad with no evidence of SARS-CoV-2 infection served as healthy controls (EAcon, *n* = 501). Questionnaires were used to assess load and duration of infectious symptoms, other complaints, exercise tolerance, and duration of training interruption at baseline and at follow-up 6 months after baseline. Diagnostic tests conducted at baseline included resting and exercise electrocardiogram (ECG), echocardiography, spirometry, and blood analyses.

**Results:**

Most acute and infection-related symptoms and other complaints were more prevalent in cNEA than in cEAs. Compared to cEAs, EAcon had a low symptom load. In cNEAs, female athletes had a higher prevalence of complaints such as palpitations, dizziness, chest pain, myalgia, sleeping disturbances, mood swings, and concentration problems compared to male athletes (*p* < 0.05). Until follow-up, leading symptoms were drop in performance, concentration problems, and dyspnea on exertion. Female athletes had significantly higher prevalence for symptoms until follow-up compared to male. Pathological findings in ECG, echocardiography, and spirometry, attributed to SARS-CoV-2 infection, were rare in infected athletes. Most athletes reported a training interruption between 2 and 4 weeks (cNEAs: 52.9%, cEAs: 52.4%), while more cNEAs (27.1%) compared to cEAs (5.1%) had a training interruption lasting more than 4 weeks (*p* < 0.001). At follow-up, 13.8% of cNEAs and 9.9% of cEAs (*p* = 0.24) reported their current exercise tolerance to be under 70% compared to pre-infection state. A persistent loss of exercise tolerance at follow-up was associated with persistent complaints at baseline, female sex, a longer break in training, and age > 38 years. Periodical dichotomization of the data set showed a higher prevalence of infectious symptoms such as cough, sore throat, and coryza in the second phase of the pandemic, while a number of neuropsychiatric symptoms as well as dyspnea on exertion were less frequent in this period.

**Conclusions:**

Compared to recreational athletes, elite athletes seem to be at lower risk of being or remaining symptomatic after SARS-CoV-2 infection. It remains to be determined whether persistent complaints after SARS-CoV-2 infection without evidence of accompanying organ damage may have a negative impact on further health and career in athletes. Identifying risk factors for an extended recovery period such as female sex and ongoing neuropsychological symptoms could help to identify athletes, who may require a more cautious approach to rebuilding their training regimen.

**Trial Registration Number:**

DRKS00023717; 06.15.2021—retrospectively registered.

**Supplementary Information:**

The online version contains supplementary material available at 10.1007/s40279-023-01976-0.

## Key Points

**Table:** 

Elite athletes do experience less symptom frequency compared to recreational athletes, with a shift towards female sex showing longer symptom duration and frequency.
Persisting exercise intolerance as observed in 14% of recreational athletes and 10% of elite athletes at 9-month follow-up is associated to the initial symptom load at baseline.
In COVID-19 infected athletes, the spectrum of symptoms is paralleled only to a small extent by pathological findings in the diagnostic workup.

## Introduction

An acute infection with SARS-CoV-2 is associated with a wide range of clinical manifestations. Although athletes are not considered a high-risk group for severe or critical cases of COVID-19, they can still experience moderate to severe symptoms, leading to the need for refraining from training and competitions [1–3]. Furthermore, recent studies have reported the possibility of cardiac involvement, specifically myocardial and myopericardial inflammation, with a prevalence estimated to be between 0.5 and 3.0% [1, 4–6]. Currently, recommendations regarding the screening of athletes for safe return to sports after SARS-CoV-2 infection and the duration to interrupt training and competitive activities are primarily based on expert opinions and lack sufficient data-driven evidence [7]. Consequently, there exists a research gap concerning the potential health risks faced by athletes when resuming their usual sports activities after recovering from COVID-19.

Another unanswered question pertains to the duration of impaired exercise tolerance and athletic performance following SARS-CoV-2 infection. Exertional dyspnea has been identified as one of the persistent symptoms lasting beyond 2 weeks after COVID-19 diagnosis [8]. COVID-19 patients may experience various lung pathologies, including broncho-obstruction, decreased respiratory muscle strength, fibrosis, and impaired diffusion capacity [9]. Additionally, even in mild cases of COVID-19, alterations on the erythrocyte level occur, and may be a factor, compromising exercise capacity [10]. It is important to acknowledge that even minor functional pathologies can limit athletes’ exercise capacity and performance. Besides cardiac and respiratory abnormalities, symptoms such as fatigue and those of possibly originating from neuropsychiatric factors, such as muscle pain, headaches, sleep disturbances, brain fog, cognitive impairment, and mental fatigue, may also hinder exercise tolerance and readiness for competitive sports. However, it remains unclear whether athletes with no or only mild acute symptoms are at risk for “ongoing symptomatic COVID-19” or even a “Post-COVID-19 Syndrome” [11]. Although the prevalence of a prolonged course of COVID-19, characterized by symptoms persisting longer than 12 weeks, has been reported to be 10% among acutely infected patients, there is currently no data available regarding long-term consequences, specifically for well-trained athletes [12].

Overall, our current understanding of the potential health consequences faced by competitive athletes infected with SARS-CoV-2 remains incomplete. Therefore, our consortium has initiated a multicenter, prospective, observational cohort study known as CoSmo-S (*COVID-19 in elite sports—A multi-center cohort study*). This study aimed to assess the pattern and duration of symptoms in athletes infected by SARS-CoV-2, collect data from cardiopulmonary and laboratory diagnostic tests, and evaluate the impact of the infection on the duration of training interruption, self-reported exercise tolerance, and performance. Additionally, we investigated whether there are any differences in these parameters between squad athletes and recreational athletes as well as between males and females. Furthermore, we aimed to analyze the clinical course of the infection through follow-up assessments and identify baseline symptoms that may indicate a persistent loss in self-reported exercise tolerance in the medium term.

## Material and Methods

### Design and Setting of the Study

**Fig. 1:**
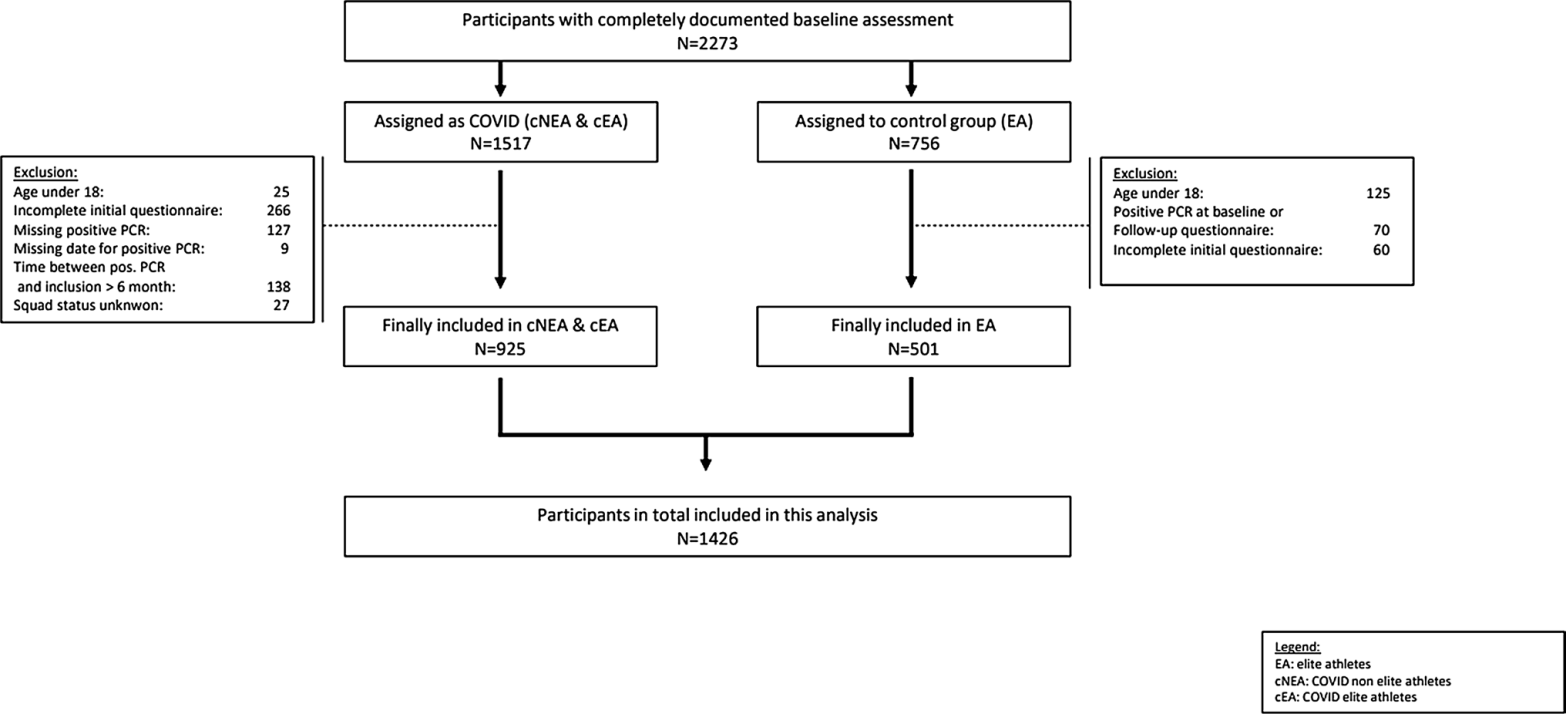
Flow chart of the recruitment process in this study. *cNEA* non elite athletes with COVID-19, *cEA* elite athletes with COVID-19, *EAcon* non-infected elite athletes

**Fig. 2:**
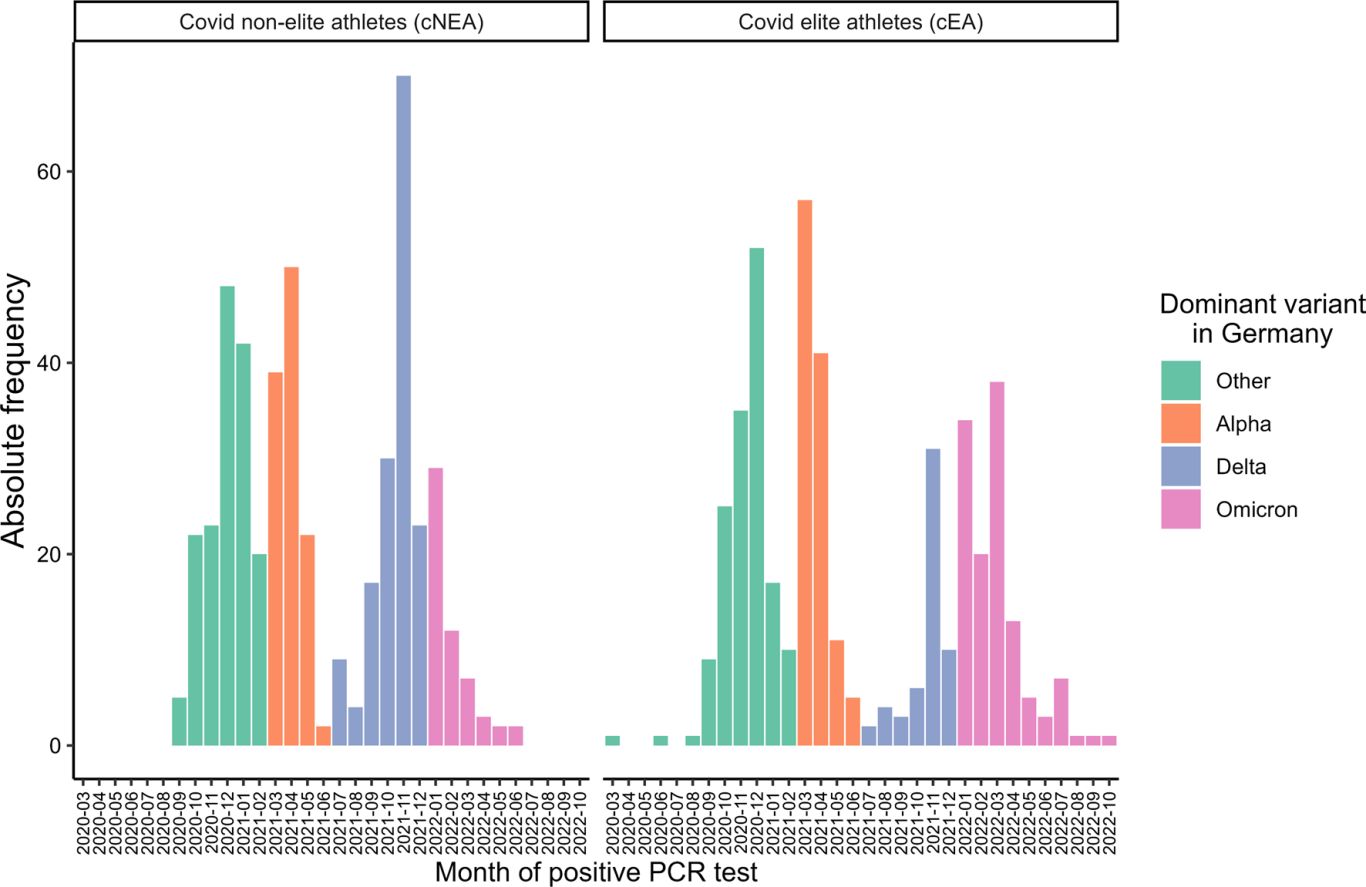
Inclusion histogram depicting the dominant variant in Germany and time of positive PCR of the athletes

Between 7 August 2020 and 28 October 2022 (see Online Supplementary Material (OSM) Fig. 1) two groups of athletes in 13 sports medical outpatient clinics in Germany were recruited: (1) Federal squad and/or professional athletes (COVID elite athletes, cEAs) and COVID non-elite athletes (cNEAs), who tested positive for SARS-CoV-2 by PCR and presented themselves as outpatients to assess eligibility for competitive sports and/or to clarify persistent symptoms or exercise intolerance after infection with SARS-CoV-2. The recruitment period was characterized by the dominance of different viral variants (Fig. 2). As healthy controls: (2) Federal squad athletes with no evidence of SARS-CoV-2 infection, who routinely present themselves for their annual sports medical preparticipation screening offered by the German Olympic Association (DOSB), served as healthy controls (healthy elite athletes control: EAcon) (Fig. 1). Further inclusion criteria for the COVID-19 group were age 18 years or older and for the squad athlete group age 14 years or older, but for the study aims presented here only data from participants who were 18 years or older went into analysis. In cNEAs, ambiguous sport activities with a minimum of three training sessions corresponding to a minimum weekly energy expenditure of 20 MET hours was a prerequisite for inclusion in the study. In the current analyses, we included athletes whose baseline visit was no more than 6 months after a positive PCR test. For the follow-up assessment, only questionnaires that were completed between 5 and 9 months after the baseline visit were considered. This time-span allowed us to monitor athletes up to 6 months after acute infection with SARS-CoV-2 and a further 9 months for their follow-up investigation. A more detailed description of the design and methods of data collection in CoSmo-S has been published elsewhere [13].

### Data Collection

After obtaining written informed consent, the athletes underwent a comprehensive evaluation encompassing their medical and sports history, a clinical examination, cardiopulmonary diagnostics, and blood sampling. Questionnaires (see OSM) were utilized to document essential information such as the type of sport, training volume, squad membership, number of training days missed, as well as specific details regarding SARS-CoV-2 associated symptoms and their duration. Additionally, the athletes were requested to assess their exercise tolerance as actually perceived (“My exercise tolerance ("*How well do I tolerate exercise*"*) currently corresponds to xx% the condition before the Covid-19 infection*”) relative to their pre-infection state on a scale ranging from 10%, indicating the worst, to 100%, representing the best. Furthermore, the athletes self-reported their actual exercise performance (“*How would you rate your current athletic form?”)* as “good,” “satisfactory,” or “bad.” For the purpose of follow-up, digital questionnaires were distributed to the participants via a hyperlink to an online survey. Email reminders were sent 2 weeks prior to the anticipated 6-month follow-up time point, at the calculated 6-month mark, and 2 weeks thereafter if participants had not responded to either survey.

In cEAs and cNEAs, the diagnostic protocol was based on the early recommendations for return to sport after SARS-CoV-2 infection in Germany [14]. In EAcon, the preparticipation screening program of the DOSB was applied. Resting 12-lead electrocardiogram (ECG) was recorded and interpreted on the basis of the criteria for athletes [15]. Exercise-ECG was obtained during an incremental exercise test. Transthoracic echocardiography was performed according to current guidelines [16]; pulmonary function was assessed by spirometry using an established protocol [17]. Venous blood samples were collected and analyzed in a certified laboratory at each study center. An extension of diagnostics such as cardiac magnetic resonance imaging (cMRI), chest computer tomography, or additional blood testing was conducted by decision of each study center when individually indicated.

### Data Management and Statistics

A fully encrypted transfer of all data to the data capture system REDCap (Research Electronic Data Capture) hosted centrally for the consortium was performed at every study center [18, 19]. Access to patient data and data entry was restricted to the respective study center by means of data access groups corresponding to the study centers. Statistical analysis was performed with the statistical software R version 4.2.1 (R Core Team, 2020, R Foundation for Statistical Computing, Vienna, Austria) and RStudio Version 2.3.492 (2022) (RStudio Team, 2019 RStudio: Integrated Development for R. RStudio, Inc., Boston, MA, USA). For quantitative data, means and standard deviations are presented, and categorical variables are summarized by absolute and relative frequencies. For comparisons between athlete groups, the focus was on differences between elite athletes who tested positive for SARS-CoV-2 (cEAs) and non-elite athletes who tested positive for SARS-CoV-2 (cNEAs), and on differences between elite athletes who tested positive for SARS-CoV-2 (cEAs) and elite athletes without a positive test (EAcon), pairwise comparisons between these groups were conducted. Binary logistic regression models were fitted to the data to compare symptom frequencies and frequencies for binary diagnostic outcomes obtained from resting or exercise ECG, from echocardiography or from laboratory data between athlete groups adjusted for age, sex, and sports type (endurance, sprint/speed, other). For continuous diagnostic outcomes (resting heart rate and maximum heart rate in exercise ECG), corresponding linear regression models were fitted to the data. For comparison of symptom frequencies between male and female athletes within cNEA, cEA, and Eacon groups, logistic regression models with the symptom of interest as dependent variable and sex as well as age and sports type as independent variables were fitted to the data. Two-sample *t* tests without adjustment for further covariates were performed for comparisons of spirometry data, as age-, sex-, and height-specific values were considered. Comparison of mean age between recruitment periods (March 2020–September 2021 vs. October 2021–October 2022) was performed using a *t* test for independent samples, and frequencies of categorical data (sex, vaccination status, symptoms) were compared between participants recruited in these two periods using chi-square tests. Chi-square tests were also used to compare frequencies of athletes who interrupted their training for more than 1 month after their positive PCR test between cEA and cNEA groups and to compare frequencies of athletes with self-reported impaired exercise tolerance at follow-up between these groups.

Logistic regression models were fitted to the data to investigate associations between training break (< 2 vs. ≥ 2 weeks) and other participant characteristics (sex, age [18–28 vs. 28–38 vs. > 38 years], training volume [< 10 vs. > 10 h per week], sports type [speed/sprint vs. endurance vs. other], resting heart rate at baseline [< 60 vs. 60–70 vs. > 70 beats per minute]) with impaired physical performance at follow-up. Odds ratios with corresponding 95% confidence intervals are presented to quantify the strength of associations. To test for associations between duration of certain symptoms and presence of impaired physical performance, trend tests were applied. All statistical tests were performed two-sided with a significance level of *α* = 5%. Due to the exploratory character of the study, no adjustment for multiple testing was performed.

### Ethics and Quality Aspects

The Ethics Committee of the Medical Faculty, University of Tübingen approved the study (reference number: 608/2020BO1). The study was carried out in accordance with the Declaration of Helsinki for experiments in humans. Written informed consent was collected from all participating athletes. To ensure valid data transfer and documentation, we implemented internal monitoring, which included randomly organized visits to single study centers for checking validity of the data transfer. The study has been registered in the German Clinical Trials Register (DRKS00023717).

## Results

### Athlete Characteristics

A total of 925 athletes with confirmed SARS-CoV-2 infection were included in our analyses at baseline. Relevant baseline characteristics are displayed in Table 1. On average, these visits took place 8 weeks after positive PCR. These athletes were divided into the cNEA (*n* = 444) and cEA (*n* = 481) groups. Additionally, the EAcon group consisted of 501 healthy squad athletes. On average, cEAs were 10.6 years younger than cNEAs. In the cNEA group, the predominant sports type was endurance (62.1%), while 71.3% of cEAs could be categorized into sprint/speed sports. In EAcon, a similar percentage of sprint/speed (44.3%) and endurance athletes (40.7%) was represented. Of the cNEA group, 83.1% reported a training volume below 10 h per week, whereas 78.1% of cEAs reported a training volume of 10 h or more per week. The most apparent comorbidity within both cNEAs (9.1%) and cEAs (7.5%) was bronchial asthma. However, asthma prevalence may have been underestimated as it was only assessed in anamnesis and not confirmed by comprehensive pulmonary diagnostics. Around one-third of the athletes in each of the groups reported allergies.

**Table 1:** Baseline characteristics of the study population

Variables	COVID non-elite athletes (cNEAs) *n* = 481	COVID elite athletes (cEAs) *n* = 444	Healthy elite athletes (EAcon) *n* = 501
Age (years) [*n* = 481/444/501]	34.3 ± 10.6	23.7 ± 5.1	23.3 ± 6.0
BMI (kg⋅m^−2^) [*n* = 470/427/492]	23.5 ± 2.9	23.8 ± 3.1	23.2 ± 3.1
Male [*n* = 474/443/495]	289 (61.0%)	286 (64.6%)	303 (61.2%)
Squad status [*n* = − /404/471]			
Olympic squad	–	37 (9.2%)	55 (11.7%)
PK, TK, EK	–	114 (28.2%)	215 (45.6%)
Professional	–	145 (35.9%)	–
Paralympic squad	–	8 (2.0%)	9 (1.9%)
NK1, NK2	–	100 (24.8%)	192 (40.8%)
Sport type [*n* = 448/414/472]			
Endurance	278 (62.1%)	97 (23.4%)	192 (40.7%)
Sprint/speed	123 (27.5%)	295 (71.3%)	209 (44.3%)
Power sport	27 (6.0%)	11 (2.7%)	20 (4.2%)
Cognition	5 (1.1%)	7 (1.7%)	41 (8.7%)
Other	15 (3.3%)	4 (1.0%)	10 (2.1%)
Training volume (h/week) [*n* = 437/434/484]			
< 5	172 (39.4%)	9 (2.1%)	18 (3.7%)
5–10	191 (43.7%)	86 (19.8%)	54 (11.2%)
10–15	59 (13.5%)	183 (42.2%)	137 (28.3%)
> 15	15 (3.4%)	156 (35.9%)	275 (56.8%)
*Comorbidities*			
Myocarditis [*n* = 447/388/462]	5 (1.1%)	3 (0.8%)	4 (0.9%)
Asthma [*n* = 452/389/463]	41 (9.1%)	29 (7.5)	31 (6.7%)
Elevated blood pressure [*n* = 449/389/462]	23 (5.1%)	12 (3.1%)	9 (1.9%)
Allergies [*n* = 446/385/460]	166 (37.2%)	103 (26.8%)	130 (28.3%)
Nutritional habits [*n* = 414/390/404]			
Normal	351 (84.8%)	360 (92.3%)	358 (88.6%)
Vegetarian + lacto-ovo-vegetarian	43 (10.4%)	22 (5.6%)	33 (8.1%)
Vegan	15 (3.6%)	6 (1.5%)	13 (3.2%)
Carbohydrate reduced	5 (1.2%)	2 (0.5%)	0 (0.0%)
Nutritional supplement intake [*n* = 455/425/486]	145 (31.9%)	195 (45.9%)	236 (48.6%)

With the exclusion of age and body mass index (BMI), data are presented as absolute numbers and %. Athletes, competing in the Bundesliga, German championships or being a professional are clustered as professional

*PK* perspective squad, *TK* team sports squad, *EK* supplementary squad, *NK1* young talent squad 1, *NK2*, young talent squad 2

### Acute and Infection-Related Symptoms

**Fig. 3:**
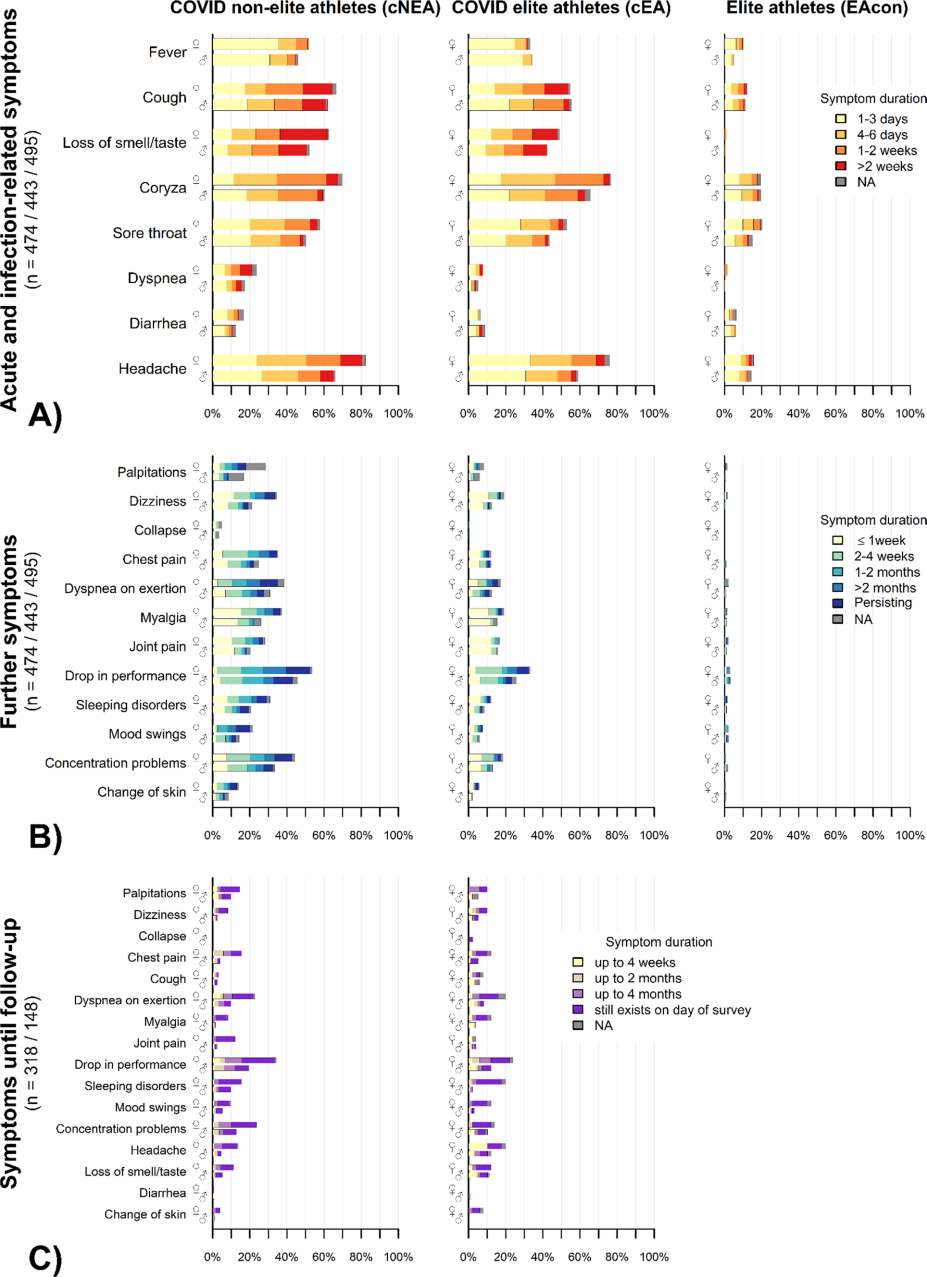
Frequency and duration of infectious (**A**), further (**B**) and follow-up symptoms (**C**) in the male and female study groups. Symptoms are presented in the group of non-elite (left, cNEA) and elite athletes (middle, cEA), tested positive for SARS-CoV-2 as well as for A and B in non-infected control elite athletes (right, EAcon). *p* values obtained through *t*-test comparing cNEA versus cEA and EA versus cEA are presented in Tables 4–9 in the supplements

At baseline, 1.5% of cNEAs and in 6.3% cEAs reported no symptoms. Leading acute and infection-related symptoms were headache, coryza, cough, a loss of smell and taste, sore throat, and fever (Fig. 3A; Table 2). With exception of coryza, diarrhea, and headache, acute and infection-related symptoms were significantly more prevalent in cNEAs compared to cEAs (*p* < 0.05). For most acute and infection-related symptoms, their duration was longer in cNEAs than in cEAs. Compared to cEAs, symptom prevalence was low in EAcon.

**Table 2:** Prevalence of acute and infection-related symptoms at baseline according to athlete groups

Acute and infection-related symptoms	COVID non-elite athletes (cNEAs) [*n* = 481]	COVID elite athletes (cEAs) [*n* = 444]	Healthy elite athletes (EAcon) [*n* = 501]	*p* value	
				cNEA vs. cEA	EAcon vs. cEA
No symptoms	7 (1.5%)	28 (6.3%)	275 (55.7%)	< 0.001	< 0.001
Fever	233 (48.4%)	150 (33.8%)	34 (6.8%)	0.04	< 0.001
Cough	306 (63.6%)	244 (55.0%)	58 (11.6%)	0.03	< 0.001
Loss of smell/taste	270 (56.1)	198 (44.6%)	4 (0.8%)	0.03	< 0.001
Coryza	309 (64.2%)	308 (69.4%)	98 (19.6%)	0.68	< 0.001
Sore throat	256 (53.2%)	208 (46.8%)	85 (17.0%)	0.003	< 0.001
Dyspnea	94 (19.5%)	27 (6.1%)	5 (1.0%)	< 0.001	< 0.001
Diarrhea	67 (13.9%)	35 (7.9%)	30 (6.0%)	0.21	0.30
Headache	350(72.8%)	288 (64.9%)	75 (15.0%)	0.12	< 0.001

Data are presented in absolute numbers and %. Group comparisons with logistic regression models adjusted for sex, age, and sports type

Compared to men, female cNEAs experienced significantly more often a loss of smell and taste, coryza, and headache (*p* < 0.05), while in cEAs only the latter two symptoms showed significant sex differences. Nine participants (1.1%) were hospitalized for a duration of 7 days or shorter, while one participant required longer hospitalization.

### Further Symptoms

A drop in performance was the leading further symptom in both groups of infected athletes, followed by concentration problems, dyspnea on exertion, and myalgia in cNEAs, and myalgia, joint pain, dizziness, and concentration problems in cEAs (Fig. 3B and Table 6 in OSM). These and all further symptoms were more prevalent in cNEAs compared to cEAs. For most further symptoms, their duration was longer in cNEAs than in cEAs. Compared to cEAs, EAcon reported no (syncope) or very few symptoms.

A significantly higher prevalence of further symptoms in females was restricted to cNEAs. In this group, palpitations, dizziness, chest pain, myalgia, sleeping disorders, mood swings, and concentration problems were significantly more prevalent in women compared to men (*p* < 0.05).

### Diagnostic Findings

At the baseline assessment, resting heart rate determined by ECG was higher in cNEAs (65.3 ± 11.9 beats·min^−1^) compared to cEAs (59.3 ± 11.3 beats·min^−1^, *p* < 0.001), but not significantly different between cEAs and EAcon (*p* = 0.16) (Table 3).

**Table 3:** Cardiopulmonary diagnostic findings

Variables	COVID non-elite athletes (cNEAs) *n* = 481	COVID elite athletes (cEAs) *n* = 444	Healthy elite athletes (EAcon) *n* = 501	*p* value	
				cNEA vs. cEA	EAcon vs. cEA
*Resting electrocardiogram (ECG)*^a^					
Resting heart rate (1 ·min^−1^) [*n* = 465/425/482]	65.3 ± 11.9	59.3 ± 11.3	58.2 ± 10.4	< 0.001	0.16
Arrhythmias [*n* = 451/392/449]	6 (1.3%)	3 (0.8%)	8 (1.8%)	0.31	0.63
Negative T-wave(s) [*n* = 468/427/482]	40 (8.5%)	63 (14.8%)	94 (19.5%)	0.04	0.04
Pathological (according international criteria) [*n* = 442/380/403]	8 (1.9%)	9 (2.4%)	10 (2.5%)	0.27	0.68
*Exercise ECG*^a^					
Arrhythmias [*n* = 450/392/445]	21 (4.7%)	12 (3.1%)	7 (1.6%)	0.83	0.12
Maximum heart rate (1 ·min^−1^) [*n* = 462/413/473]	181.1 (13.3)	184.4 (12.8)	185.9 (13.0)	0.002	0.15
*Echocardiography*^a^					
LV enlargement [*n* = 467/426/467]	21 (4.5%)	32 (7.5%)	51 (10.9%)	0.08	0.13
LV systolic dysfunction					
Ejections fraction decreased [*n* = 102/280/259]	9 (8.8%)	28 (10.0%)	11 (4.2%)	0.90	0.20
Fractional shortening decreased [*n* = 449/403/432]	71 (15.8%)	36 (8.9%)	36 (8.3)	0.005	0.86
LV diastolic dysfunction [*n* = 405/343/396]	1 (0.2%)	0 (0.0%)	1 (0.3%)	§	§
RV systolic dysfunction [*n* = 421/399/431]	3 (0.7%)	2 (0.5%)	8 (1.9%)	0.23	0.59
Wall motion abnormalities [*n* = 458/404/456]	1 (0.2%)	0 (0.0%)	1 (0.2%)	§	§
Pericardial effusion [*n* = 449/413/457]	1 (0.2%)	4 (1.0%)	3 (0.7%)	0.28	0.99
*Spirometry*^b^					
FVC % predicted	101.8% ± 14.2%	107.3% ± 14.0%	107.1% ± 14.3%	< 0.001	0.84
Decreased [*n* = 440/402/414]	24 (5.5%)	10 (2.5%)	8 (1.9%)	0.02	0.58
FEV1% predicted	101.8% ± 12.9%	105.4% ± 13.7%	104.5% ± 14.4%	< 0.001	0.37
Decreased [*n* = 448/407/415]	20 (4.5%)	14 (3.4%)	20 (4.8%)	0.38	0.33
FEV1/VC % predicted	104.1% ± 16.1%	101.0% ± 12.1%	100.3% ± 10.2%	0.001	0.43
Decreased [*n* = 442/403/413]	12 (2.7%)	21 (5.2%)	23 (5.6%)	0.06	0.82

Data are presented as absolute numbers and %. Data are presented as means ± standard deviation or as absolute and relative frequencies. Categorial data: Left ventricle was considered enlarged if LV end-diastolic diameter/body surface area was > 31 mm/m^2^ (males) or > 30 mm/m^2^ (females). LV systolic function was defined as decreased if EF was < 52% for men or < 54% for women or FS < 25%. LV diastolic function was considered impaired if *e*/*e*′ was > 13 (lateral), > 15 (septal), or > 14 (average of lateral and septal measurement). RV function was considered impaired if tricuspid annular plane systolic excursion (TAPSE) was < 17 mm. FVC, FEV1, and FEV1/FVC were considered impaired if the absolute value was measured below the 5% percentile. Moreover, spirometric data are presented in %predicted

*LV* left ventricular, *RV* right ventricular, *FS* fractional shortening, *EF* ejection fraction (Simpson), *FVC* forced vital capacity, *FEV1* forced expiratory volume in 1 s

^a^Group comparisons with linear regression models for continuous and logistic regression models for binary outcomes adjusted for sex, age, and sports type

^b^Group comparisons with t tests for independent samples or chi-square tests

In EAcon and cEAs, negative T-waves occurred more frequently (19.5/14.8%), but most of them did not meet the criteria to be abnormal in an athletic population [20]. According to the International Guidelines for the athlete’s ECG [20], only 2.4% of cEAs and 1.9% of cNEAs exhibited pathological findings, and no significant differences were found between the groups. In the exercise ECG, arrhythmias occurred in 4.7% (cNEAs) and 3.1% (cEAs) and 1.6% (EAcon) of the athletes; single premature ventricular and supraventricular beats were not rated.

The percentages of abnormal echocardiographic findings were 0.2% (cNEAs)/1.0% (cEAs) for pericardial effusion, 0.2/0.0% for wall motion abnormalities, 0.7%/0.5% for right ventricular (RV) systolic dysfunction and 0.2/0.0% for left ventricular (LV) diastolic dysfunction, respectively, with no relevant differences between cEA and EAcon. LV systolic function as assessed by fractional shortening (FS) was slightly reduced in some athletes, with a higher proportion of impaired FS observed in cNEAs than in cEAs, but no differences were observed between cEAs and cNEAs. In contrast, ejection fraction (EF) was decreased more frequently in cEAs (10.0%) compared to EAcon (4.2%), but this difference was not statistically significant after adjustment for sex, age, and sports type (*p* = 0.20). With a very few exceptions, additional diagnostics in cEAs with decreased EF did not show pathological findings suspicious for myocardial damage (Table 11, OSM). In total, 41 (4.4%) of the infected athletes were sent for additional cMRI; in four (9.8%) of them signs of cardiac involvement (late gadolinium enhancement, edema, pericardial effusion) could be detected. The remaining athletes did not have findings suspicious of cardiac injury in cMRI.

In all three groups, the variables of resting spirometry FVC, FEV1, and FEV1/FVC showed largely normal values when expressed in percent of predicted values. Similarly, the percentage of athletes with a FVC, FEV1, or FEV1/FVC below the 5% percentile was low.

Only few athletes showed an elevation of plasma C-reactive protein (CRP) levels in cNEAs and cEAs with no relevant differences as compared to EAcon (Table 4). The percentage of creatinine, GOT (glutamic-oxaloacetic transaminase), lactate dehydrogenase (LDH), and creatinine kinase (CK) levels above the upper limit were significantly higher in cEAs compared to cNEAs (*p* < 0.05), but did not differ relevantly between cEAs and EAcon. Compared to cEAs (2.8%), more cNEAs (10.8%) exhibited elevated levels of plasma ferritin (*p* = 0.01).

**Table 4:** Laboratory findings in venous blood samples

Variables	COVID non-elite athletes (cNEAs) (*n* = 481)	COVID elite athletes (cEAs) (*n* = 444)	Healthy elite athletes (EAcon) (*n* = 501)	*p* value	
				cNEA vs. cEA	EAcon vs. cEA
*Blood cell counts*					
Erythrocytes decreased [*n* = 463/405/472]	14 (3.0%)	15 (3.7%)	16 (3.4%)	0.41	0.31
Hemoglobin decreased [*n* = 463/407/469]	28 (6.0%)	21 (5.2%)	27 (5.8%)	0.4	0.96
Neutrophils [*n* = 453/364/424]					
Decreased	38 (8.4%)	14 (3.8%)	30 (7.1%)	0.02	0.19
Elevated	8 (1.8%)	3 (0.8%)	10 (2.4%)	0.15	0.07
Lymphocytes [*n* = 453/368/429]					
Decreased	20 (4.4%)	6 (1.6%)	23 (5.4%)	0.02	0.04
Elevated	15 (3.3%)	10 (2.7%)	6 (1.4%)	0.85	0.16
*Serum/plasma analyses*					
C-reactive protein elevated [*n* = 416/341/209]	7 (1.7%)	7 (2.1%)	8 (3.8%)	0.99	0.4
GOT elevated [*n* = 420/348/312]	22 (5.2%)	47 (13.5%)	47 (15.1%)	0.003	0.92
GPT elevated [*n* = 463/400/449]	40 (8.6%)	43 (10.8%)	32 (7.1%)	0.55	0.06
LDH elevated [*n* = 97/188/146]	5 (5.2%)	39 (20.7%)	27 (18.5%)	0.02	0.83
Gamma-GT elevated [*n* = 423/390/460]	13 (3.1%)	12 (3.1%)	5 (1.1%)	0.45	0.14
Urea elevated [*n* = 459/396/471]	11 (2.4%)	11 (2.8%)	19 (4.0%)	0.81	0.49
Creatinine elevated [*n* = 464/406/473]	13 (2.8%)	34 (8.4%)	44 (9.3%)	0.01	0.29
Creatine kinase elevated [*n* = 463/407/466]	90 (19.4%)	146 (35.9%)	211 (45.3%)	0.004	< 0.001
Troponin (I or T) elevated [*n* = 381/249/84]	5 (1.3%)	15 (6.0%)	4 (4.8%)	0.02	0.58
Ferritin elevated [*n* = 427/399/457]	46 (10.8%)	11 (2.8%)	17 (3.7%)	0.01	0.52

Data are presented as absolute numbers and %. Group comparisons with logistic regression models adjusted for sex, age and sports type

*GOT* glutamic oxaloacetic transaminase, *GPT* glutamic oxaloacetic transaminase, *gamma-GT* gamma glutamyl transferase, *LDH* lactate dehydrogenase

### Symptoms Until Follow-Up

Half of cNEA (50.5%) and more than half of cEA (56.4%) were free of symptoms during the period until and at follow-up (p = 0.77) (Fig. 3C and Table 7 in OSM). Drop in performance (25.1%/16.1%), concentration problems (17.0%/11.4%), and dyspnea on exertion (14.6%/12.1%) were the leading symptoms that were reported in the follow-up questionnaire. Other than for acute and infection-related symptoms, frequencies of reported symptoms did not differ significantly between cNEAs and cEAs when comparisons were adjusted for age, sex, and sports type. Regarding the occurrence of symptoms until follow-up, for seven symptoms in cNEAs and three in cEAs this was significantly higher in female compared to male athletes (see Fig. 3C and Table 10 in OSM).

### Interruption of Training and Self-Reported Exercise Tolerance and Performance

Compared to cEAs (5.1%), cNEAs reported more often (27.1%) having an interruption of training of more than 1 month (*p* < 0.001, chi-square test) (Fig. 2, OSM). In both groups, half of the athletes paused their training for 2–4 weeks. Only 1.4% of the cEAs did not interrupt their training. Among athletes who started exercising again within 1–2 weeks after infection, no-one reported fever at this time point, while in a few cases further acute and infection-related symptoms such as cough (4.8%), coryza (2.2%), or headache (1.8%) still existed after a return to training.

**Fig. 4:**
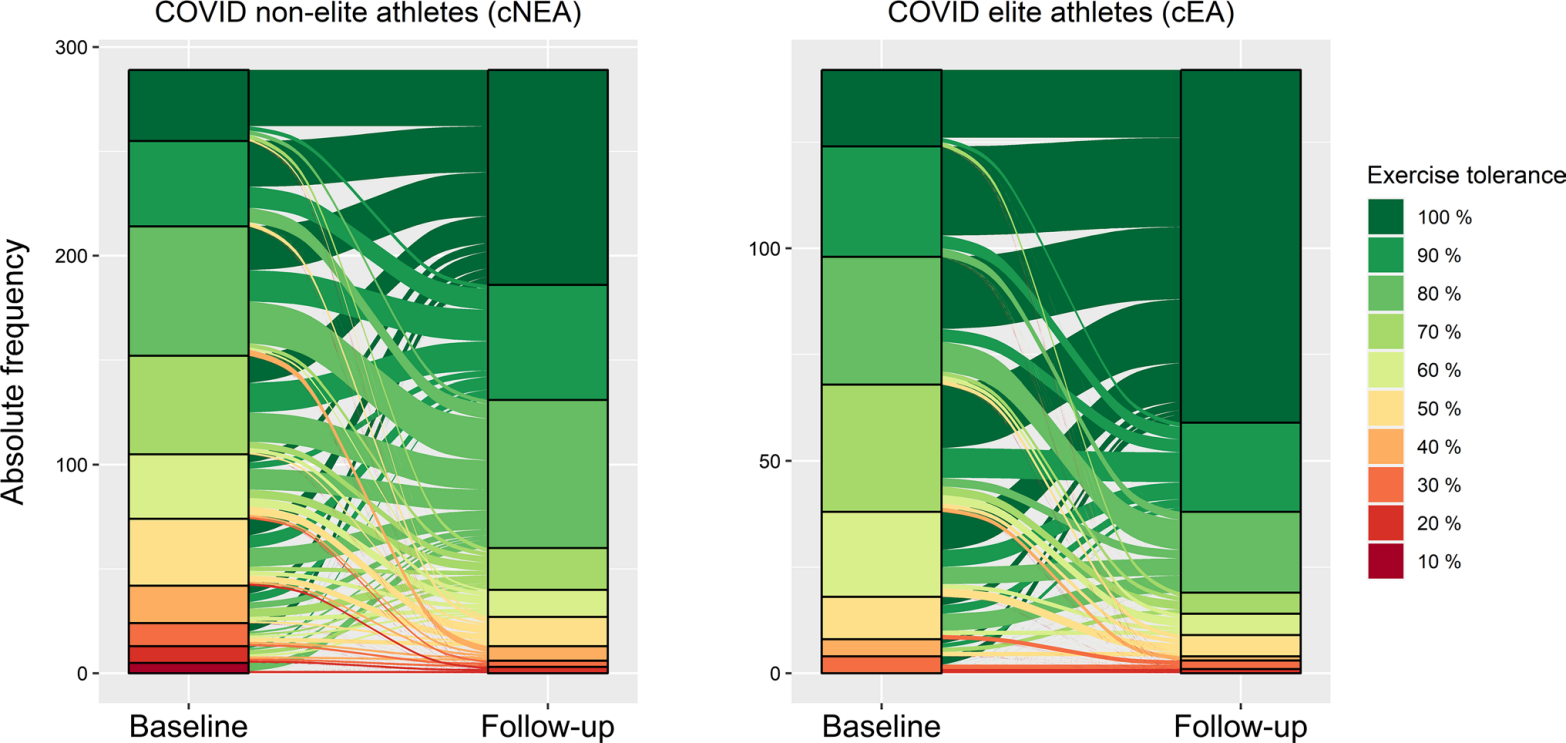
Alluvial plot illustrating self-reported exercise tolerance as mentioned by the athletes at baseline and follow-up in cNEA (left) and cEA (right). Bars illustrate absolute frequencies of athletes rating their exercise tolerance at 10, …, 100%. Links between the bars indicate the numbers of individuals moving from baseline categories to the given follow-up categories

At baseline, 37.3% of cNEAs and 21.3% of cEAs reported their current exercise tolerance at a level under 70% compared to their pre-infection state. At follow-up, still 13.8% of cNEAs and 9.9% of cEAs remained below this level (*p* = 0.24, chi-square test, Fig. 4). At follow-up, a questionnaire regarding self-reported exercise performance was answered by 89.1% of the cEAs as “*good*” or “*satisfactory*” compared to 69.1% in this group at baseline. Thus, at follow-up, cEAs almost reached the corresponding results of healthy EAcon (93.2%).

### Predictors of Self-Reported Exercise Tolerance at Follow-Up

**Fig. 5:**
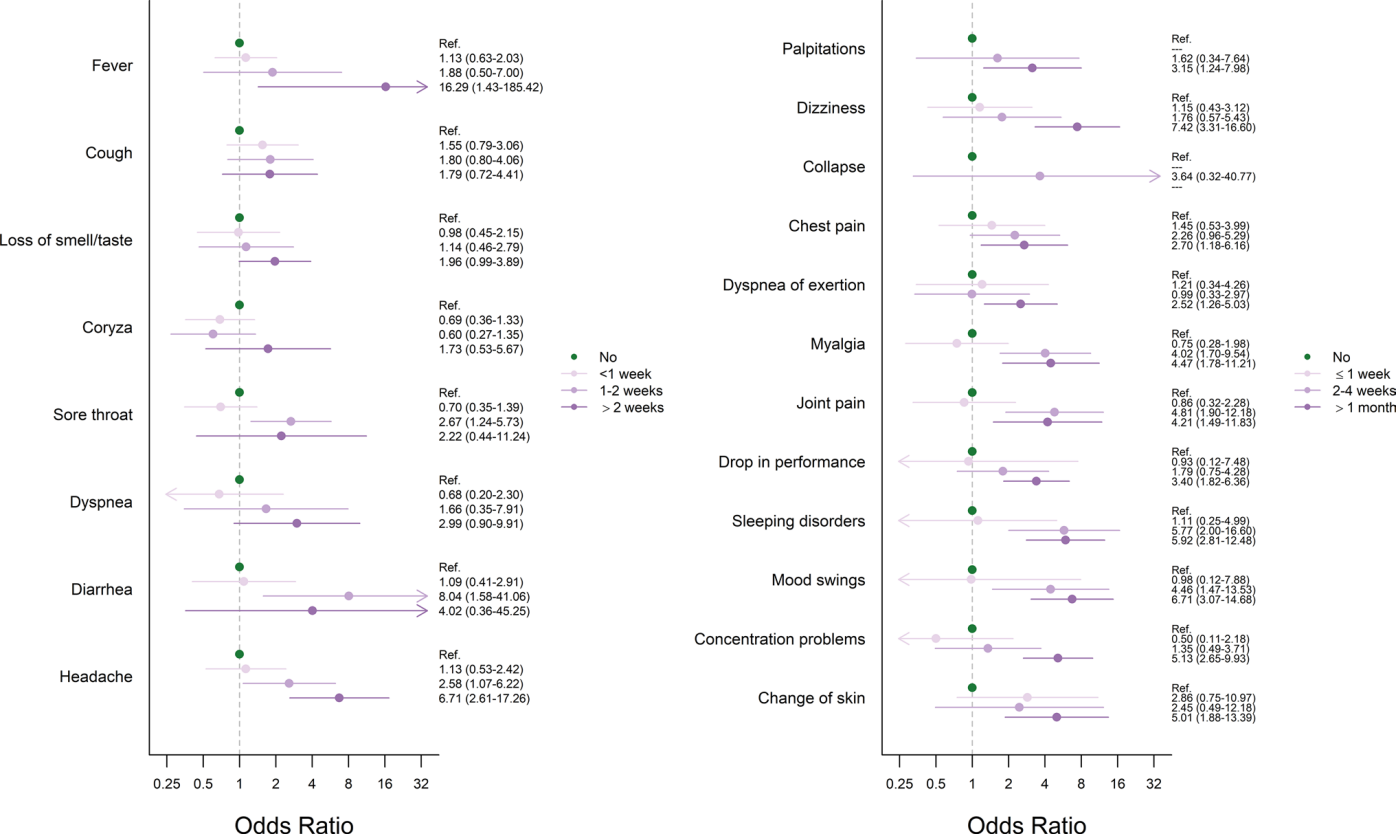
Odds ratios and associated 95% confidence intervals for the risk of a self-reported exercise tolerance below 70% compared to pre-infection state (= 100%) at follow-up for each infectious (left) and further symptom (right) and its duration at baseline

A logistic regression analysis investigating potential predictors for impaired exercise tolerance reported in the follow-up questionnaire revealed an association between symptom duration at baseline and a persistent reduction of self-reported exercise tolerance below 70% of the pre-infection state at follow-up (Fig. 5). A significant relationship was observed between reduced exercise tolerance and duration of the acute and infectious symptoms diarrhea and headache. Moreover, a higher risk for reduced exercise tolerance at follow-up was found for individuals who reported longer persistence of palpitations, chest pain, dyspnea on exertion, mood swings, concentration problems, sleeping disturbances, skin changes, dizziness, joint pain, and myalgia (Fig. 5). Female athletes (odds ratio (OR) male vs. female: 0.44, 95% confidence interval (CI) 0.25–0.78), participants aged > 38 years (> 38 years vs. 18–28 years: OR 2.03, 95% CI 1.05–3.95; 29–38 years vs. 18–28 years: OR 0.84, 95% CI 0.39–1.76), and athletes with a training break of more than two weeks > 2 weeks versus ≤ 2 weeks, (OR 3.41, 95% CI 1.53–9.07) had a higher risk for decreased exercise tolerance at follow-up. For training volume (> 10 h per week vs. < 10 h per week, OR 0.83, 95% CI 0.44–1.49), sports type (speed/sprint vs. endurance OR 0.96, 95% CI 0.52–1.76; other vs. endurance, OR 1.21, 95% CI 0.43–2.94), and resting heart rate at baseline (60–70 bpm vs. < 60 bpm, OR 1.34, 95% CI 0.69–2.63; > 70 vs. < 60 bpm, OR 1.79, 95% CI 0.89–3.59) no significant association was observed.

### Periodical Data Analyses

Periodical dichotomization of the data set by the date of PCR testing (March 2020–September 2021 vs. October 2021–October 2022) revealed differences in the prevalence of symptoms but also in vaccination state (Table 5). Cough, sore throat, and coryza were significantly more frequent during the second phase of the pandemic, while loss of taste and smell, fever, dyspnea on exertion, myalgia, joint pain, mood swings, and concentration disorders were reported in a lesser amount during this time (*p* < 0.05). No significant differences were found for self-reported exercise tolerance as well as for all other symptoms at follow-up.

**Table 5:** Recruiting period clustered into two timespans

	March 2020–September 2021 [*n* = 577]	October 2021–October 2022 [*n* = 348]	*p* value
Age, years	29.5 (10.6)	28.8 (8.9)	0.33
Male	360 (62.9%)	215 (62.3%)	0.85
Vaccinated	38 (6.6%)	293 (84.2%)	< 0.001
*Acute and infection-related symptoms*			
No symptoms	22 (3.8%)	13 (3.7%)	0.95
Fever	254 (44.0%)	129 (37.1%)	0.04
Cough	324 (56.2%)	226 (64.9%)	0.01
Loss of smell/taste	325 (56.3%)	143 (41.1%)	< 0.001
Coryza	365 (63.3%)	252 (72.4%)	0.004
Sore throat	265 (45.9%)	199 (57.2%)	< 0.001
Dyspnea	88 (15.3%)	33 (9.5%)	0.01
Diarrhea	76 (13.2%)	26 (7.5%)	0.01
Headache	408 (70.7%)	230 (66.1%)	0.14
*Further symptoms*			
No further symptoms	137 (23.7%)	93 (26.7%)	0.31
Palpitations	93 (16.1%)	39 (11.2%)	0.04
Dizziness	132 (22.9%)	61 (17.5%)	0.05
Collapse	13 (2.3%)	9 (2.6%)	0.75
Chest pain	130 (22.5%)	63 (18.1%)	0.11
Dyspnea on exertion	158 (27.4%)	66 (19.0%)	0.0041
Myalgia	161 (27.9%)	61 (17.5%)	< 0.001
Joint pain	133 (23.1%)	52 (14.9%)	0.003
Drop in performance	236 (40.9%)	126 (36.2%)	0.16
Sleeping disorders	110 (19.1%)	53 (15.2%)	0.14
Mood swings	82 (14.2%)	29 (8.3%)	0.01
Concentration problems	170 (29.5%)	75 (21.6%)	0.01
Change of skin	51 (8.8%)	17 (4.9%)	0.03
*Symptoms until follow-up*	[*n* = 332]	[*n* = 140]	
No further symptoms	166 (50.0%)	81 (57.9%)	0.12
Palpitations	32 (9.6%)	15 (10.7%)	0.72
Dizziness	18 (5.4%)	8 (5.0%)	00.85
Collapse	1 (0.3%)	1 (0.7%)	0.19
Chest pain	31 (9.3%)	8 (5.7%)	0.48
Dyspnea on exertion	47 (14.2%)	18 (12.9%)	0.71
Myalgia	16 (4.8%)	7 (5.0%)	0.93
Joint pain	19 (5.7%)	8 (5.7%)	0.99
Drop in performance	72 (21.7%)	33 (23.6%)	0.65
Sleeping disorders	40 (12.0%)	10 (7.1%)	0.11
Mood swings	23 (6.9%)	8 (5.7%)	0.63
Concentration problems	57 (17.2%)	15 (10.7%)	0.08
Headache	33 (9.9%)	15 (10.7%)	0.80
Loss of smell/taste	33 (9.9%)	8 (5.7%)	0.14
Diarrhea	2 (0.6%)	2 (1.4%)	0.37
Change of skin	8 (2.4%)	3 (2.1%)	0.86

Prevalence of acute and infection-related as well as further symptoms at baseline and symptoms that occurred or were still present until follow-up (FU) according to both periods. Data are presented in absolute numbers and %. Group comparisons were conducted using the *t*-test for independent samples (age) or chi-square test (sex, vaccination status, symptoms)

## Discussion

To the best of our knowledge, our multicenter study is the first to examine the effects of SARS-CoV-2 infection in a large cohort of recreational and elite athletes including non-infected controls over a follow-up period of nearly a year, which included the assessment of sex differences. We demonstrated that the pattern of reported acute and infection-related symptoms of a SARS-CoV-2 infection is quite consistent with that in recently published studies in the general population [21, 22] and in athletes [1, 2, 6, 23–26]. Most of the athletes experienced mild or moderate symptoms at baseline and very few participants required hospitalization. Intensive care was not necessary in any cases. A number of complaints were more prevalent in cNEAs than in cEAs, and female sex was partly associated with a higher symptom load. Pathological findings in our diagnostic procedures were rare in the infected athletes. Most athletes reported a training interruption of between 2 and 4 weeks. Moreover, continued reduced self-reported exercise tolerance at follow-up was associated with the prevalence and duration of a number of symptoms at baseline.

The finding that frequency and duration of symptoms such as cough, dyspnea on exertion, anosmia/dysgeusia, as well as others, were significantly higher in cNEA is new and differs from the results of Lemes and his group, who did not report differences in the severity of symptoms between studies in professional/elite and college/university athletes [23]. As there was a significant and on average 10-year difference in age between the cEA and cNEA group, one may conclude that our finding is potentially age dependent. However, the lower percentage of symptoms in cEAs compared to cNEAs remains stable after adjusting for age. This is in line with recent findings from the pre-COVID-19 era, which showed that a high training volume is associated with a lower number of self-reported illness days during an upper respiratory tract infection [27]. Whether this result may be due to an effect of high training loads on immune function and/or a selection bias remains to be determined.

An important finding already observed by others in the general population [22] as well as in athletes [28] was that a number of infectious and further symptoms were more frequent among women compared to men. However, with the exception of coryza and headache, this sex difference was restricted to cNEAs. The reasons for the higher reported symptom prevalence in non-elite women remain unclear and reflect an important area of future research.

In contrast to clinical symptoms, results of further diagnostics revealed mainly normal findings. Interpretation of resting ECG based on the current recommendations for athletes yielded no relevant differences in the percentage of pathological findings in elite athletes (2.4%) and the control group (2.5%). Compared to controls, we did not observe higher resting heart rates after SARS-CoV-2 infection in cEAs, as reported in an earlier study [29]. A similar picture emerges analyzing echocardiography results. Pathological findings typical for SARS-CoV-2 cardiac involvement such as pericardial effusion and regional wall motion abnormalities were only observed in very few cases in our cohort. A significantly higher percentage of cEAs with decreased EF compared to the healthy EAcon was further clarified by additional diagnostics, including global longitudinal strain and no evidence of myocardial dysfunction was found. Thus, subclinical LV systolic dysfunction as described in normal subjects recovering from SARS-CoV-2 infection seems to be rather improbable [30]. These findings are in accordance with other studies in athletes reporting similar rates of abnormalities in ECG and echocardiography [1, 6, 31, 32]. In the study of Martinez et al., 19 of 789 professional athletes exhibited echocardiographic signs indicative of cardiac injury [31]. Similar to recent reports [1, 31], with a proportion of 0.4% in the entire cohort, only few of the infected athletes exhibited signs of myocardial injury in the cMRI [1, 31]. However, a cMRI was only ordered in small proportion of the infected athletes, consequently subclinical cases of myocarditis cannot be ruled out.

Taken together, our findings are in line with previous reports, which suggest that cardiac sequelae after infection with SARS-CoV-2 among athletes seems to be less frequent than assumed in the initial phase of the pandemic. Similar to others [1, 23, 33], no severe cardiac events have been reported up to now in our study population. However, a remaining risk of an acute myocardial involvement in athletes with SARS-CoV-2 infection must be kept in mind and long-term effects on the heart cannot be excluded.

Our spirometric data showed no evidence of a functional impairment, and we mainly observed results of FVC, FEV1 and FEV1/FVC above the lower level on normal values. The small percentage of athletes with spirometric results below the lower limit of normal ranges (fifth percentile) did not differ relevantly from our control group of healthy squad athletes. No evidence of spirometric impairments were reported in competitive athletes [25] but also in a larger number of young healthy adults [34]. In contrast, Rasmusen et al. found an obstructive lung function or radiological signs of COVID-19 in 15 of 122 elite athletes after SARS-CoV-2 infection [33]. It cannot be ruled out that a more comprehensive pulmonary diagnostic procedure including the assessment of diffusion capacity and respiratory muscle strength may reveal abnormal results relevant for exercise performance. Moreover, we cannot exclude that even subtle reductions in lung function have relevant negative effects on performance in elite athletes at the individual level.

The laboratory results revealed more athletes with elevated plasma ferritin in cNEAs compared to cEAs (10.8 vs. 2.8%). Ferritin is an acute phase biomarker and hyperferritinemia has been shown to be predictive for a more severe disease and poor outcome [35]. Whether the elevated ferritin levels in some cNEAs were the result of their SARS-CoV-2 infection remains unclear, and further factors such as their higher age may play a role. The remaining laboratory results did not show any other signs of systemic inflammation. Other laboratory findings with different percentages of athletes with analytical results outside the reference values do not seem to be caused by the SARS-CoV-2 infection and appear to be subject to sport-specific variations.

Thus, the broad spectrum of symptoms is echoed only to a small extent by pathological findings of the diagnostic workup. We cannot exclude that pathological findings may have been missed because baseline diagnostics took place on average 8 weeks after the positive PCR. Nevertheless, infections with SARS-CoV-2 have a relevant impact on complete recovery and full recovery of exercise tolerance or good performance even in cEAs, as half of the athletes in this group had to take a break from training for 2–4 weeks and an additional 5.1% had to interrupt exercising for longer than 1 month. In 147 international competing athletes, mainly from Great Britain and Northern Island, 14% of the athletes lost more than 28 days until full training and competition participation [2]. In other cohorts, the median duration of return to training after COVID-19 ranged from 14 to 30 days [24, 26, 28, 32], which may be due to different predominant viral variants, a variable vaccination state, and a younger age [28]. In our study, the higher percentage of athletes coming back “soon” after SARS-CoV-2 infection compared to cNEAs was potentially due to their milder symptoms during the infection. A greater motivation of cEAs to return to training and competition may, however, also play a role. In this context it is important to note that only a small proportion of athletes reported to have started exercise with mild symptoms like cough, headache, or coryza.

As a finding of concern, there is a relevant number of athletes still reporting complaints such as a drop in performance, exertional dyspnea, palpitations, sleep disturbances, mood swings, concentration problems, and joint pain as well as a reduced exercise tolerance at follow-up. Similar to our results, post-acute symptoms lasting several weeks have also been reported in a meta-analysis of [23], while in other studies, 10–15% of the athletes suffered from complaints for longer than 12 weeks after infection [25, 32]. We add to these findings with the aspect that well-trained athletes are also at risk for the persistence of complaints in a time frame exceeding half a year. Moreover, even in elite athletes, symptoms seem to persist more often in females compared to men, a finding we already know from the general population [22].

Our trend analyses revealed a significant relationship between the duration of several symptoms as assessed at baseline and fittingly the duration of the training break with the reduced exercise tolerance at follow-up, particularly if the symptoms lasted longer than 1 month. Interestingly, nearly half of the symptoms analyzed to be predictive regarding a reduced exercise tolerance at follow-up could be classified as neuropsychiatric. From a clinical perspective, special guidance including a further monitoring of complaints should be provided to these athletes during their return to sport. Other predictor analyses [2, 24] found in symptomatic athletes that dyspnea reported in the acute phase of SARS-CoV-2 infection was indicative of an exceeded loss of training time (> 28 days) [2, 24]. In another study the prevalence of long COVID symptoms during a median follow-up of 107 days post-infection was predicted by symptoms in the acute phase of infection [32]. Another factor associated with decreased exercise tolerance at follow-up in our study was female sex and to a lesser extent age over 38 years, whereas resting heart rate, sports type and training volume did not appear to have a relevant effect. Our analyses comparing different time periods of the pandemic yielded infected athletes with a partially different symptom pattern. Typical infection symptoms such as cough, sore throat, and coryza were more prevalent in the second phase, while a number of neuropsychiatric symptoms as well as dyspnea on exertion were less frequent in the later phase of the pandemic. Although there were significant differences in symptoms between the two periods, these were not extreme, restricted to the baseline assessments, and do not alter our main conclusions. Moreover, it was not possible to work out the respective influence of the different virus variants and vaccination state on the individual course of infection.

## Limitations

Our study has some limitations that may have impacted the findings and should be considered when interpreting our results. Our cohort was confined to infected athletes, presenting themselves to outpatient clinics on their own initiative, and who may not be representative of the overall athlete population in Germany. There was a longer period between the positive test and baseline data collection, which may hinder the detection of short-term pathological findings. Moreover, the study suffers from only incomplete control data from the pre-COVID-19 era, not allowing detection of subtle changes on the individual level. Especially with respect to our follow-up surveys, we cannot exclude that infections other than COVID-19 may have influenced our findings. We are also aware that data from questionnaires are subjective and at risk of being prone to bias. Nevertheless, we think that if exercise tolerance is perceived as poor from an athlete, it may affect his or her performance, and is therefore relevant information. Our project suffers from the known limitations of multicenter observational studies, which include some heterogeneity across our study centers with respect to the diagnostic yield. In addition, we lost some athletes at follow-up, which could affect our findings. And finally, the influence of vaccination history and different virus variants on our partly variable results during the entire recruitment period could not be addressed in more detail, as both factors changed in parallel.

## Conclusion

Compared to recreational athletes, elite athletes seem to be at lower risk of being or remaining symptomatic after SARS-CoV-2 infection. It remains to be determined whether persistent complaints after SARS-CoV-2 infection without evidence of accompanying organ damage may have a negative impact on further health and career in athletes. Identifying risk factors for an extended recovery period such as female sex and ongoing neuropsychiatric symptoms could help to identify athletes who may require monitoring and a more cautious approach to rebuilding their training regimen.

### Supplementary Information

Below is the link to the electronic supplementary material.

Supplementary file1 (PDF 592 kb)

## Declarations

### Data availability

All data supporting the findings of this study are available within the paper and its Online Supplementary Material.
