# Supplementary material for: COVID-19 in Female and Male Athletes: Symptoms, Clinical Findings, Outcome, and Prolonged Exercise Intolerance—A Prospective, Observational, Multicenter Cohort Study (CoSmo-S)
Source: Sports Med. 2024 Jan 11;54(4):1033–49. doi: 10.1007/s40279-023-01976-0 (PMC11052799; doi:10.1007/s40279-023-01976-0)
Supplement: Supplementary file 1 — Supplementary file1 (PDF 592 kb) [file 40279_2023_1976_MOESM1_ESM.pdf]

Springer - Sports Medicine

COVID-19 in female and male athletes: Symptoms, clinical findings outcome and prolonged exercise intolerance — A prospective observational multi-center cohort study (CoSmo-S)

COVID-19 in elite sports – symptoms and reduced exercise intolerance

Manuel Widmann[1]*, Roman Gaidai[2], Isabel Schubert[1], Maximilian Grummt[3], Lieselotte Bensen[3], Arno Kerling[4], Anne Quermann[5], Jonas Zacher[6], Shirin Vollrath[7], Daniel Alexander Bizjak[7], Claudia Beckendorf[8], Florian Egger[9], Erik Hasler[10], Klaus-Peter Mellwig[11], Cornelia Fütterer[12], Fritz Wimbauer[13], Azin Vogel[13], Julia Schoenfeld[13], Jan C. Wüstenfeld[3,15], Tom Kastner[3,15], Friedrich Barsch[14], Birgit Friedmann-Bette[5], Wilhelm Bloch[16], Tim Meyer[9], Frank Mayer[8], Bernd Wolfarth[3,15], Kai Roecker[10], Claus Reinsberger[2], Bernhard Haller[12#] and Andreas M. Niess[1#]

1 Department of Sports Medicine, Medical University Hospital Tuebingen, Tuebingen 72072, Germany
2 Department of Sports and Health, Institute of Sports Medicine, Paderborn University, Germany
3 Department of Sports Medicine, Charité-Universitätsmedizin Berlin and Humboldt-Universität zu Berlin, Berlin, Germany
4 Department for Rehabilitation and Sports Medicine, Hannover Medical School, 30559 Hannover, Germany
5 Medical Clinic VII, Department of Sports Medicine, University Hospital Heidelberg, Heidelberg, Germany
6 Institute of Cardiology and Sports Medicine, Department of Preventative and Rehabilitative Sports and Performance Medicine, German Sports University Cologne, Cologne, Germany.
7 Division of Sports and Rehabilitation Medicine, Ulm University Medical Center, Ulm, Germany
8 University of Potsdam, Outpatient Clinic, Center of Sports Medicine, Germany
9 Institute of Sports and Preventive Medicine, Saarland University, Saarbrücken, Germany
10 Institute for Applied Health Promotion and Exercise Medicine (IfAG), Furtwangen University, Germany
11 Clinic for General and Interventional Cardiology/Angiology, Herz- und Diabeteszentrum NRW, Ruhr-Universität Bochum, Bad Oeynhausen, Germany
12 Technical University of Munich, School of Medicine, Institute of AI and Informatics in Medicine, Munich, Germany
13 Department of Prevention and Sports Medicine, University Hospital 'rechts der Isar', Technical University of Munich, 80992 Munich, Germany
14 University Freiburg, Medical faculty, Institute of exercise and occupational medicine, Hugstetter Str. 55, 79106, Freiburg
15 Institute for Applied Training Science, Leipzig University, Leipzig, Germany
16 Department of Molecular and Cellular Sport Medicine, Institute of Cardiovascular Research and Sport Medicine, German Sport University Cologne, Cologne, Germany

Trial registration number: DRKS00023717; 06.15.2021 - retrospectively registered

# Shared last authorship
CoSmo-S consortium:

[Mr Mike Peter Birnbaum
mike-peter.birnbaum@charite.de
Department of Sports Medicine, Charité-Universitätsmedizin Berlin and Humboldt-Universität zu Berlin, Berlin, Germany

Mr Philipp Stapmanns
philip.stapmanns@charite.de
Department of Sports Medicine, Charité-Universitätsmedizin Berlin and Humboldt-Universität zu Berlin, Berlin, Germany

Mrs Franziska Greiss
franziska.greiss@charite.de
Department of Sports Medicine, Charité-Universitätsmedizin Berlin and Humboldt-Universität zu Berlin, Berlin, Germany

Mr Gerald Schneider
Schneider.Gerald@mh-hannover.de
Department for Rehabilitation and Sports Medicine, Hannover Medical School, 30559 Hannover, Germany

Mr Karsten Keller
Karsten.Keller@med.uni-heidelberg.de
Medical Clinic VII, Department of Sports Medicine, University Hospital Heidelberg, Heidelberg, Germany

Mr Hans Georg Predel
Predel@dshs-koeln.de
Institute of Cardiology and Sports Medicine, Department of Preventative and Rehabilitative Sports and Performance Medicine, German Sports University Cologne, Cologne, Germany

Mr Michael Cassel
mcassel@uni-potsdam.de
University of Potsdam, Outpatient Clinic, Center of Sports Medicine, Germany

Mrs Sarah Szekessy
sarah.szekessy@uni-potsdam.de
University of Potsdam, Outpatient Clinic, Center of Sports Medicine, Germany

Mr Andreas Venhorst
andreas.venhorst@uni-saarland.de
Institute of Sports and Preventive Medicine, Saarland University, Saarbrücken, Germany

Mr Christian Zickwolf
christian.zickwolf@uni-saarland.de
Institute of Sports and Preventive Medicine, Saarland University, Saarbrücken, Germany

Mrs Lynn Matits
lynn.matits@uni-ulm.de
Division of Sports and Rehabilitation Medicine, Ulm University Medical Center, Ulm, Germany

Mr Prof. Dr. Jürgen Michael Steinacker
Juergen.Steinacker@uniklinik-ulm.de
Division of Sports and Rehabilitation Medicine, Ulm University Medical Center, Ulm, Germany

Mr Gunnar Erz
Gunnar.Erz@med.uni-tuebingen.de
Department of Sports Medicine, Medical University Hospital Tuebingen, Tuebingen 72072, Germany

Mr Prof. Dr. Christof Burgstahler
Christof.Burgstahler@med.uni-tuebingen.de
Department of Sports Medicine, Medical University Hospital Tuebingen, Tuebingen 72072, Germany

Mrs Dr Christine Kopp
Christine.Kopp@med.uni-tuebingen.de
Department of Sports Medicine, Medical University Hospital Tuebingen, Tuebingen 72072, Germany]

Mr Martin Halle
Martin.Halle@mri.tum.de
Department of Prevention and Sports Medicine, University Hospital 'rechts der Isar', Technical University of Munich, 80992 Munich, Germany

Stephanie Zapf
Stephanie.Zapf@mri.tum.de
Department of Prevention and Sports Medicine, University Hospital 'rechts der Isar', Technical University of Munich, 80992 Munich, Germany

Katrin Esefeld
Katrin.Esefeld@mri.tum.de
Department of Prevention and Sports Medicine, University Hospital 'rechts der Isar', Technical University of Munich, 80992 Munich, Germany

Peter Rüdrich
ruedrich@iat.uni-leipzig.de
Institute for Applied Training Science, Leipzig University, Leipzig, Germany

Judith Hesse
hesse@iat.uni-leipzig.de
Department of Sports Medicine, Charité-Universitätsmedizin Berlin and Humboldt-Universität zu Berlin, Berlin, Germany
Institute for Applied Training Science, Leipzig University, Leipzig, Germany

Peter Deibert
peter.deibert@uniklinik-freiburg.de
University Freiburg, Medical faculty, Institute of exercise and occupational medicine, Hugstetter Str. 55, 79106, Freiburg

*** Corresponding author**

Manuel Widmann
Department of Sports Medicine
Medical Clinic
University Hospital of Tuebingen
Hoppe-Seyler Str. 6
72076 Tuebingen
Germany
Manuel.widmann@med.uni-tuebingen.de

<u>Supplements: Table 6:</u> Prevalence of further symptoms at baseline according to athlete groups. Data are presented in absolute numbers and %.

Further Symptoms	COVID non - elite athletes (cNEA) [n = 481]	COVID elite athletes (cEA) [n = 444]	Healthy elite athletes (EAcon) [n = 501]	p-value	
				cNEA vs. cEA	EAcon vs. cEA
No further symptoms	77 (16.0%)	153 (34.5%)	365 (72.9%)	< 0.001	< 0.001
Palpitations	102 (21.2%)	30 (6.8%)	5 (1.0%)	< 0.001	< 0.001
Dizziness	127 (26.2%)	66 (14.9%)	5 (1.0%)	0.007	< 0.001
Collapse	19 (4.0%)	3 (0.7%)	0 (0.0%)	0.004	§
Chest pain	139 (28.9%)	54 (12.2%)	3 (0.6%)	< 0.001	< 0.001
Dyspnea on exertion	161 (33.5%)	63 (14.2%)	7 (1.4%)	< 0.001	< 0.001
Myalgia	148 (30.5%)	74 (16.7%)	8 (1.6%)	0.001	< 0.001
Joint pain	114 (23.7%)	71 (16.0%)	9 (1.8%)	0.049	< 0.001
Drop in performance	236 (49.1%)	126 (28.4%)	16 (3.2%)	< 0.001	< 0.001
Sleeping disorders	120 (24.9%)	43 (9.7%)	7 (1.4%)	< 0.001	< 0.001
Mood swings	82 (17.0%)	29 (6.5%)	11 (2.2%)	0.001	0.005
Concentration problems	179 (37.2%)	66 (14.9%)	7 (1.4%)	< 0.001	< 0.001
Change of skin	53 (11.0%)	15 (3.4%)	4 (0.8%)	0.001	0.010

Group comparisons with logistic regression models adjusted for sex, age and sports type.

§ No test performed, as no event was observed in EAcon.

<u>Supplements: Table 7:</u> Prevalence of symptoms that occurred or were still present until follow-up (FU) according to athlete groups. Data are presented in absolute numbers and %.

Symptoms until FU	COVID non -elite athletes (cNEA) [n = 323]	COVID elite athletes (cEA) [n = 149]	p-value* cNEA vs. cEA
No further symptoms	163 (50.5%)	84 (56.4%)	0.77
Palpitations	37 (11.5%)	10 (6.7%)	0.07
Dizziness	15 (4.6%)	10 (6.7%)	0.07
Collapse	0 (0.0%)	2 (1.3%)	§
Chest pain	28 (8.7%)	11 (7.4%)	0.64
Cough	9 (2.8%)	10 (6.7%)	0.20
Dyspnea on exertion	47 (14.6%)	18 (12.1%)	0.84
Myalgia	13 (4.0%)	10 (6.7%)	0.47
Joint pain	21 (6.5%)	6 (4.0%)	0.74
Drop in performance	81 (25.1%)	24 (16.1%)	0.19
Sleeping disorders	38 (11.8%)	12 (8.1%)	0.79
Mood swings	22 (6.8%)	9 (6.0%)	0.56
Concentration problems	55 (17.0%)	17 (11.4%)	0.82
Headache	26 (8.0%)	22 (14.8%)	0.15
Loss of smell/taste	24 (7.4%)	17 (11.4%)	0.07
Diarrhea	3 (0.9%)	1 (0.7%)	0.73
Change of skin	7 (2.2%)	4 (2.7%)	0.69

*Comparisons adjusted for age, sex and sports type (endurance, sprint/speed, other).
§ No test performed, as no event was observed in cNEA.

<u>Supplements: Table 8:</u> Prevalence of acute and infection-related symptoms at baseline in COVID non - elite athletes (cNEA), COVID elite athletes (cEA) and healthy elite athletes (EAcon) according to gender. Data are presented in absolute numbers and %.

Acute and infection-related symptoms	Female	Male	p-value*
**cNEA**	**[n = 185]**	**[n = 289]**	
No symptoms	2 (1.1%)	5 (1.7%)	0.83
Fever	95 (51.4%)	133 (46.0%)	0.29
Cough	123 (66.5%)	179 (61.9%)	0.64
Loss of smell/taste	116 (62.7%)	150 (51.9%)	0.04
coryza	129 (69.7%)	174 (60.2%)	0.03
Sore throat	107 (57.8%)	145 (50.2%)	00.15
Dyspnea	44 (23.8%)	50 (17.3%)	0.16
Diarrhea	31 (16.8%)	36 (12.5%)	0.10
Headache	152 (82.2%)	191 (66.1%)	< 0.001
**cEA**	**[n = 157]**	**[n = 286]**	
No symptoms	3 (1.9%)	25 (8.7%)	0.01
Fever	52 (33.1%)	98 (34.3%)	0.90
Cough	86 (54.8%)	158 (55.2%)	0.92
Loss of smell/taste	77 (49.0%)	121 (42.3%)	0.16
coryza	120 (76.4%)	188 (65.7%)	0.03
Sore throat	83 (52.9%)	125 (43.7%)	0.06
Dyspnea	12 (7.6%)	15 (5.2%)	0.34
Diarrhea	10 (6.4%)	25 (8.7%)	0.53
Headache	119 (75.8%)	169 (59.1%)	< 0.001
**EAcon**	**[n = 192]**	**[n = 303]**	
No symptoms	106 (55.2%)	169 (55.8%)	0.70
Fever	19 (9.9%)	15 (5.0%)	0.01
Cough	23 (12.0%)	34 (11.2%)	0.81
Loss of smell/taste	2 (1.0%)	2 (0.7%)	0.53
Coryza	37 (19.3%)	60 (19.8%)	00.96
Sore throat	39 (20.3%)	46 (15.2%)	0.07
Dyspnea	3 (1.6%)	2 (0.7%)	0.32
Diarrhea	12 (6.2%)	18 (5.9%)	0.95
Headache	30 (15.6%)	44 (14.5%)	0.74

*Comparisons adjusted for age and sports type (endurance, sprint/speed, other).

<u>Supplements: Table 9:</u> Prevalence of further symptoms at baseline in COVID non-elite athletes (cNEA), COVID elite athletes (cEA) and healthy elite athletes (EAcon) according to gender. Data are presented in absolute numbers and %.

Further symptoms	Female	Male	p-value*
**cNEA**	**[n = 185]**	**[n = 289]**	
No further symptoms	20 (10.8%)	56 (19.4%)	0.05
Palpitations	53 (28.6%)	48 (16.6%)	0.01
Dizziness	64 (34.6%)	61 (21.1%)	0.004
Collapse	9 (4.9%)	10 (3.5%)	0.68
Chest pain	65 (35.1%)	72 (24.9%)	0.02
Dyspnea on exertion	71 (38.4%)	89 (30.8%)	0.09
Myalgia	69 (37.3%)	75 (26.0%)	0.02
Joint pain	52 (28.1%)	59 (20.4%)	0.10
Drop in performance	99 (53.5%)	132 (45.7%)	0.13
Sleeping disorders	57 (30.8%)	60 (20.8%)	0.01
Mood swings	40 (21.6%)	41 (14.2%)	0.05
Concentration problems	82 (44.3%)	96 (33.2%)	00.03
Change of skin	26 (14.1%)	25 (8.7%)	0.07
**cEA**	**[n = 157]**	**[n = 286]**	
No further symptoms	40 (25.5%)	113 (39.5%)	0.01
Palpitations	13 (8.3%)	17 (5.9%)	0.47
Dizziness	30 (19.1%)	36 (12.6%)	0.10
Collapse	1 (0.6%)	2 (0.7%)	0.99
Chest pain	19 (12.1%)	35 (12.2%)	0.98
Dyspnea on exertion	27 (17.2%)	36 (12.6%)	0.20
Myalgia	30 (19.1%)	44 (15.4%)	0.30
Joint pain	26 (16.6%)	45 (15.7%)	00.81
Drop in performance	52 (33.1%)	74 (25.9%)	0.14
Sleeping disorders	19 (12.1%)	24 (8.4%)	0.14
Mood swings	12 (7.6%)	17 (5.9%)	0.18
Concentration problems	29 (18.5%)	37 (12.9%)	0.06
Change of skin	9 (5.7%)	6 (2.1%)	0.06
**EAcon**	**[n = 192]**	**[n = 303]**	
No further symptoms	143 (74.5%)	217 (71.6%)	0.58
Palpitations	3 (1.6%)	2 (0.7%)	0.24
Dizziness	3 (1.6%)	2 (0.7%)	0.30
Collapse	0 (0.0%)	0 (0.0%)	§
Chest pain	0 (0.0%)	3 (1.0%)	§
Dyspnea on exertion	4 (2.1%)	3 (1.0%)	0.20
Myalgia	3 (1.6%)	4 (1.3%)	0.83
Joint pain	4 (2.1%)	4 (1.3%)	0.50
Drop in performance	6 (3.1%)	10 (3.3%)	0.70
Sleeping disorders	3 (1.6%)	4 (1.3%)	0.72
Mood swings	4 (2.1%)	7 (2.3%)	0.60
Concentration problems	1 (0.5%)	6 (2.0%)	0.30
Change of skin	1 (0.5%)	3 (1.0%)	0.66

*Comparisons adjusted for age and sports type (endurance, sprint/speed, other).

§ No test performed, as no event was observed in one of the groups.

<u>Supplements: Table 10:</u> Prevalence of symptoms until follow-up (FU) in COVID non - elite athletes (cNEA) as well as COVID elite athletes (cEA) according to gender. Data are presented in absolute numbers and %.

Symptoms until FU	Female	Male	p-value*
**cNEA**	**[n = 123]**	**[n = 195]**	
No further symptoms	49 (40.0%)	111 (56.9%)	0.01
Palpitations	18 (14.6%)	19 (9.7%)	0.11
Dizziness	10 (8.1%)	5 (2.6%)	0.06
Collapse	0 (0%)	0 (0%)	§
Chest pain	19 (15.4%)	8 (4.1%)	< 0.001
Cough	4 (3.3%)	5 (2.6%)	0.77
Dyspnea on exertion	28 (22.8%)	19 (9.7%)	0.003
Myalgia	10 (8.1%)	3 (1.5%)	0.01
Joint pain	15 (12.2%)	5 (2.6%)	< 0.001
Drop in performance	42 (34.1%)	38 (19.5%)	0.005
Sleeping disorders	19 (15.4%)	19 (9.7%)	0.15
Mood swings	12 (9.8%)	10 (5.1%)	0.17
Concentration problems	29 (23.6%)	25 (12.8%)	0.01
Headache	17 (13.8%)	9 (4.6%)	0.01
Loss of smell/taste	14 (11.4%)	10 (5.1%)	0.06
Diarrhea	1 (0.8%)	2 (1.0%)	0.81
Change of skin	5 (4.1%)	2 (1.0%)	0.08
**cEA**	**[n = 50]**	**[n = 98]**	
No further symptoms	21 (42.0%)	62 (63.3%)	0.02
Palpitations	5 (10.0%)	5 (5.1%)	0.18
Dizziness	5 (10.0%)	5 (5.1%)	0.18
Collapse	0 (0.0%)	2 (2.0%)	§
Chest pain	6 (12.0%)	5 (5.1%)	0.23
Cough	4 (8.0%)	6 (6.1%)	0.58
Dyspnea on exertion	10 (20.0%)	8 (8.1%)	0.03
Myalgia	6 (12.0%)	4 (4.1%)	0.05
Joint pain	2 (4.0%)	4 (4.1%)	0.92
Drop in performance	12 (24.0%)	12 (12.2%)	0.08
Sleeping disorders	10 (20.0%)	2 (2.0%)	0.003
Mood swings	6 (12.0%)	3 (3.1%)	0.03
Concentration problems	7 (14.0%)	10 (10.2%)	0.34
Headache	10 (20.0%)	12 (12.2%)	0.13
Loss of smell/taste	6 (12.0%)	11 (11.2%)	0.70
Diarrhea	0 (0.0%)	1 (1.0%)	§
Change of skin	4 (8.0%)	0 (0.0%)	§

*Comparisons adjusted for age and sports type (endurance, sprint/speed, other).
§ No calculation made, as no event was observed in one of the groups.

<u>Supplements: Table 11:</u> Additional diagnostic findings in COVID elite athletes (cEA) with decreased EF in the echocardiography (n=28).

Diagnostics	Available	Pathological	Finding
Electrocardiogram	28 (100%)	2 (7%)	Negative T-waves in V3 and V4<br>Complete right bundle block
Exercise electrocardiogram	25 (89%)	1 (4%)	Singular premature ventricular contractions
Echocardiography			
- Pericardial effusion	28 (100%)	0 (0%)	-
- Wall motion abnormalities	28 (100%)	0 (0%)	-
- Diastolic dysfunction	26 (93%)	0 (0%)	-
- Strain analysis	21 (75%)	0 (0%)	-
Troponin I or T	19 (68%)	0 (0%)	-
cMRI	4 (14%)	0 (0%)	-

EF, ejection fraction; cMRI, cardiac magnetic resonance imaging.

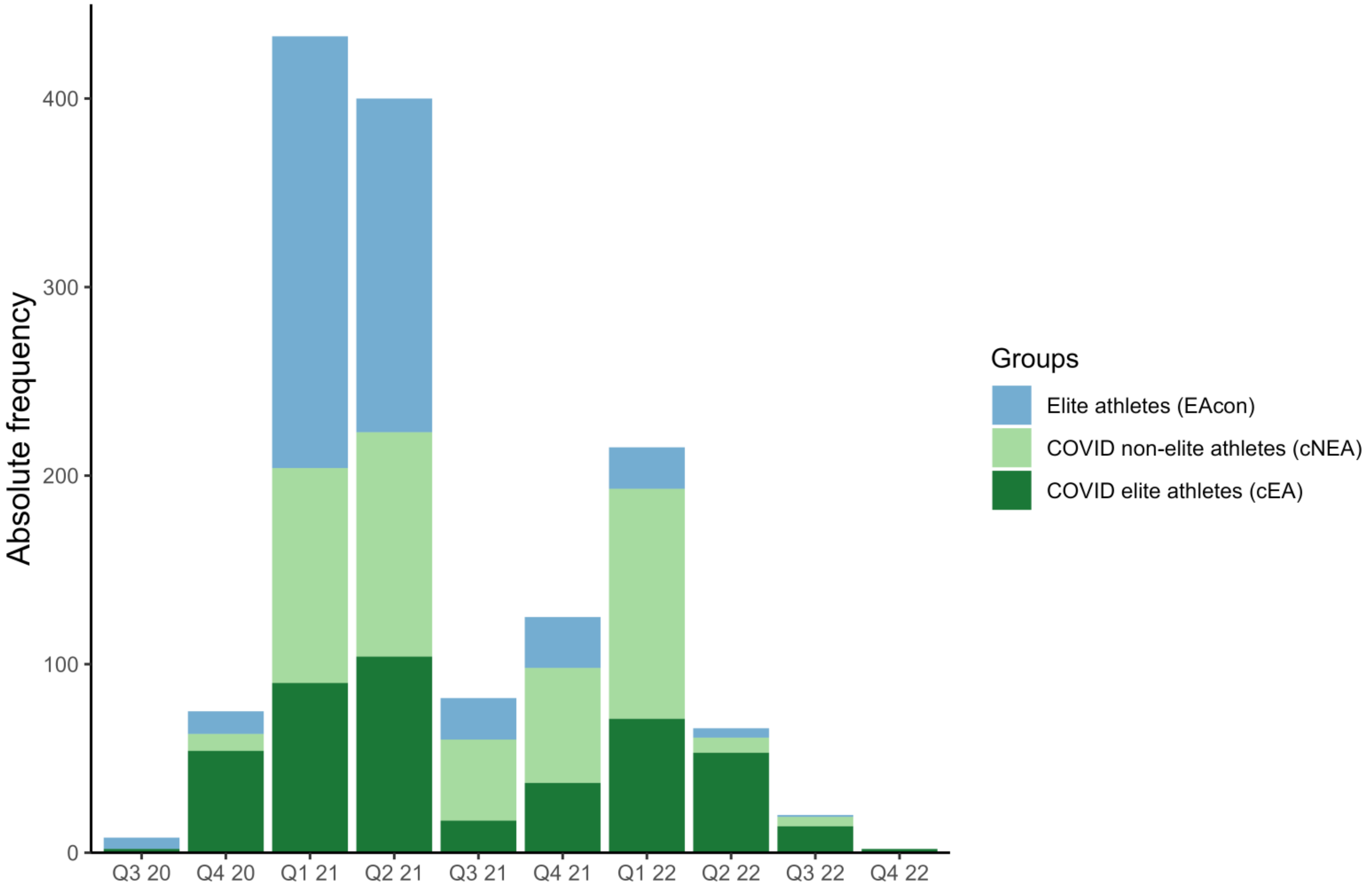

<u>Supplementary figure 1:</u> Inclusion histogram for the entire study cohort during the recruitment period from August 7th, 2020 until October 28th, 2022.

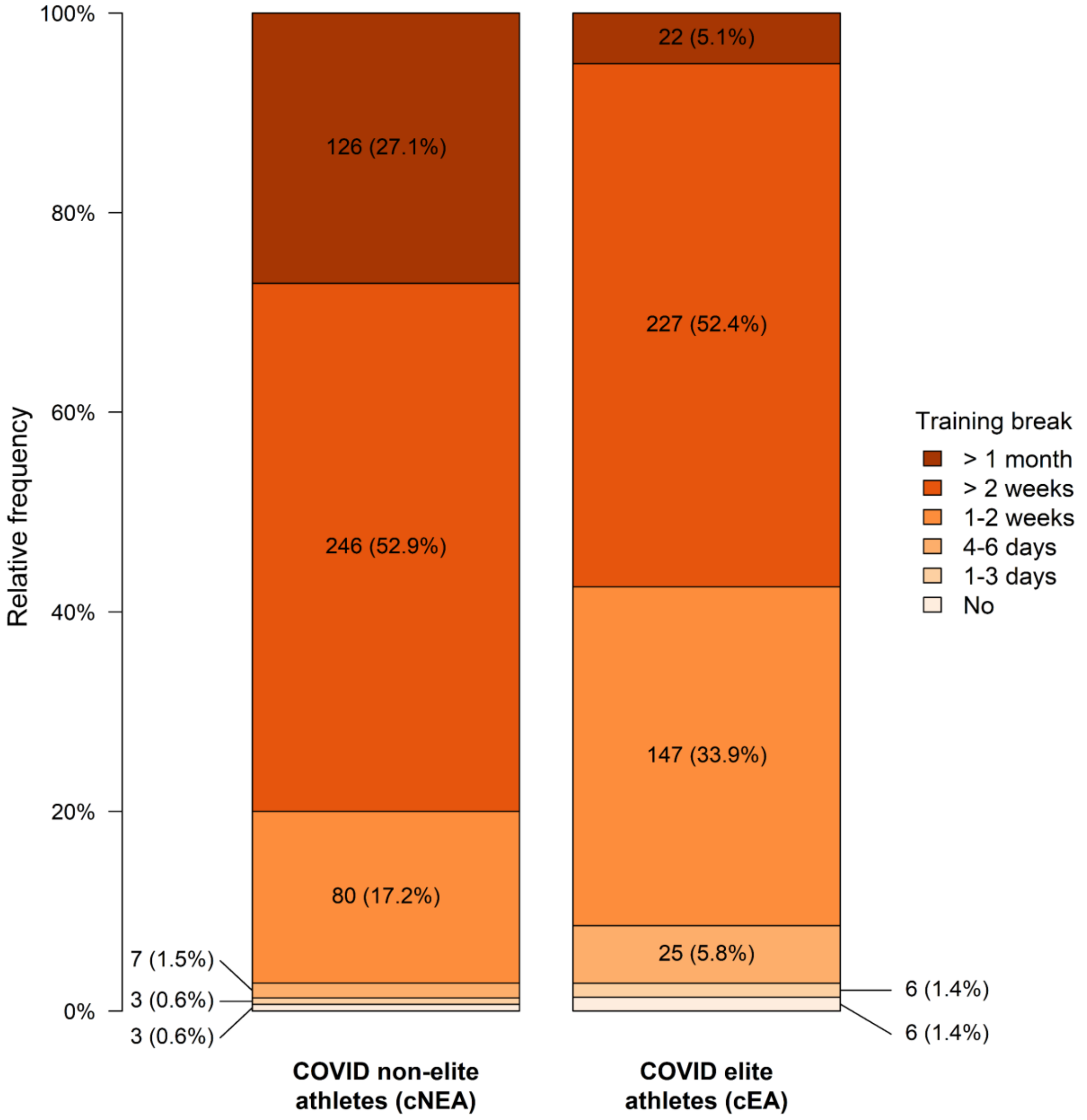

<u>Supplementary figure 2:</u> Relative frequency of training break for both COVID groups (cNEA and cEA).
